# The complete mitochondrial genome of *Dendrodoris krusensternii* (Gastropoda, Nudibranchia, Dendrodorididae) from South Korea

**DOI:** 10.1080/23802359.2024.2435915

**Published:** 2024-12-05

**Authors:** Seunghyun Lee, Seongjun Bae

**Affiliations:** Department of Ecology and Conservation, Marine Biodiversity Institute of Korea, Seocheon, South Korea

**Keywords:** Mollusca, Phyllidoidea, mitochondrial genome, phylogeny

## Abstract

The complete mitochondrial genome sequence of *Dendrodoris krusensternii* (J. E. Gray, 1850) was determined using next-generation sequencing. The complete mitochondrial genome of *D. krusensternii* is 14,361 bp long, comprising 13 genes, including 13 protein-coding genes (PCGs), 22 transfer RNAs, and two ribosomal RNAs. The nucleotide composition was estimated: 28.7% A, 14.8% C, 19.6% G, and 36.9% T. Phylogenetic analysis was performed using the maximum likelihood method (including 13 PCGs). *D. krusensternii* is related to the Phyllidiidae (superfamily Phyllidioidea), suggesting a distinct phylogenetic placement within Nudibranchia. This study represents a genomic resource, contributing to molecular studies on the evolution of the Dendrodorididae.

## Introduction

**Figure 1.**
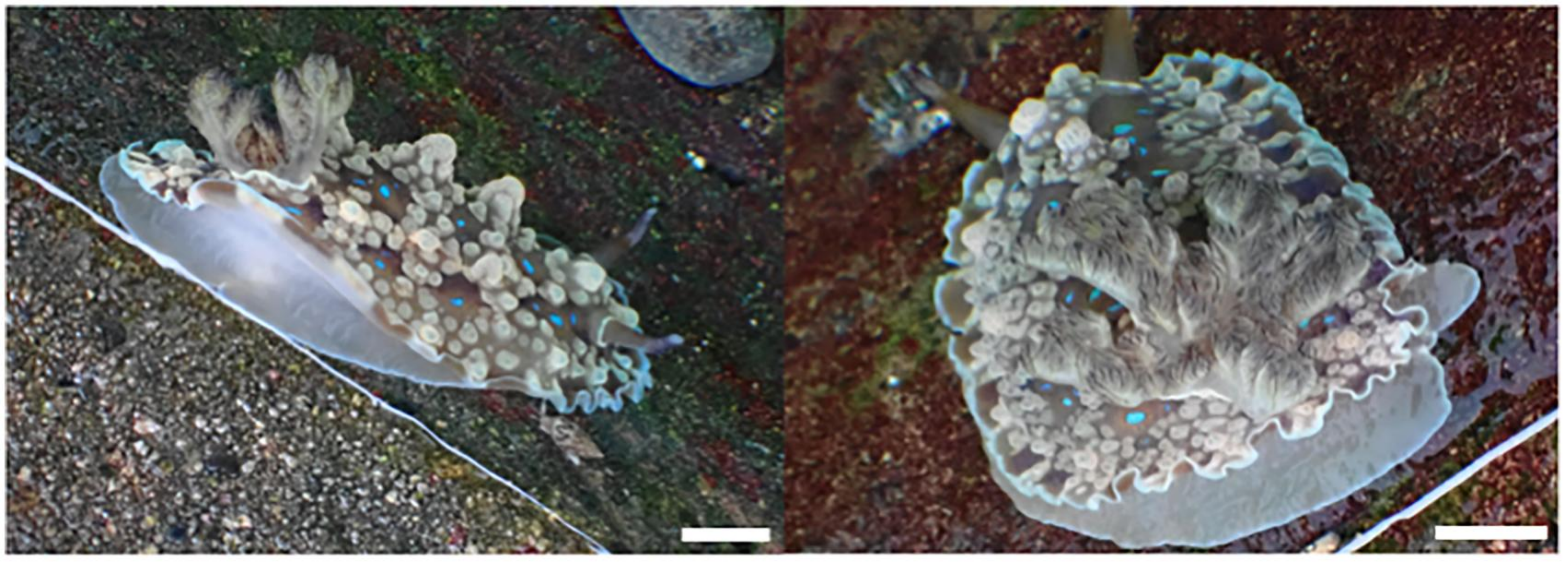
Image of the live *Dendrodoris krusensternii* (scale bar = 1 cm, photo by Seunghyun Lee).

The taxonomy of the genus *Dendrodoris* has caused considerable confusion, largely due to inadequate anatomical studies and the indiscriminate use of several characteristics to separate species (Valdès et al. 1996). Therefore, a parallel molecular phylogenetic study is important. There are 82 species of *Dendrodoris* worldwide but only seven have been reported in South Korea (MABIK (National Marine Biodiversity Institute of Korea) 2022). *Dendrodoris* is rich in species (45 species, Maniei and Wägele 2024), surpassing even *Chromodoris* (39 species, Tibiriçá et al. 2020) in the order Nudibranchia. The genus *Dendrodoris* belongs to the Dendrodorididae family, whose members are characterized by a lack of spicules in the dorsum and a radula, an important taxonomic trait for distinguishing and defining nudibranch species. *Dendrodoris krusensternii* is characterized by large, brown, and white tubercules interspersed with smooth areas and bright sky-blue spots (Khanam and Kazmi 2016; Figure 1). It is widespread in the Indo-Pacific region and is found along the entire New South Wales coast but not on Lord Howe Island. This species exhibits considerable morphological variation across its range (Nimbs and Smith 2021). Although there are many external intraspecific variable features, most studies conducted to date have primarily focused on morphological data (Valdès et al. 1996; Valdés and Gosliner 1999), except for Hirose et al. (2015) and Park et al. (2019), who used molecular data to identify Japanese and Korean species (Gali-Camps et al. 2022). Herein, we present the complete mitochondrial genome of *D. krusensternii*, providing the first report of a complete mitochondrial genome in this genus.

**Figure 2.**
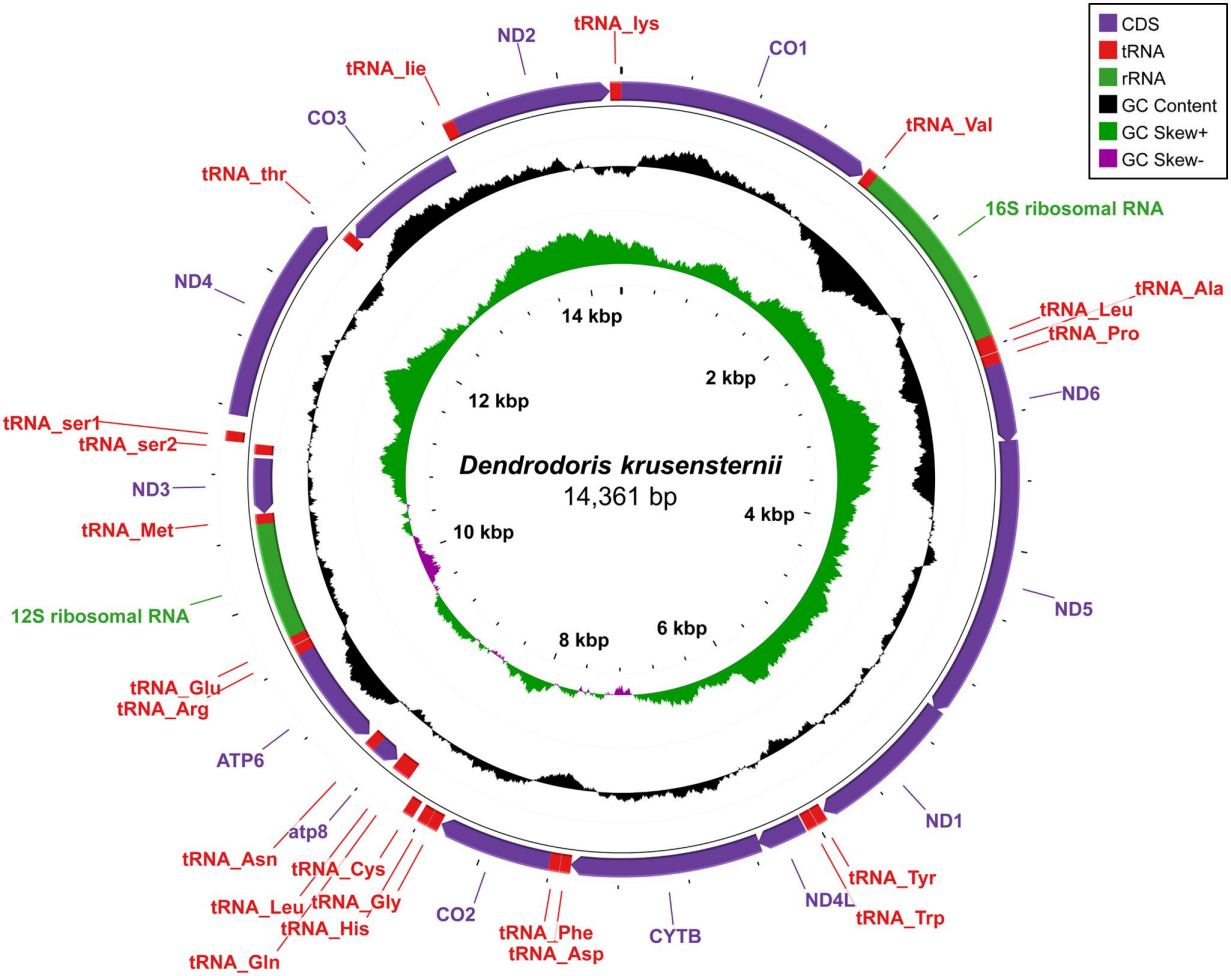
Mitochondrial circular map of *Dendrodoris krusensternii*. The map was generated with CGView server (Grant and Stothard 2008). PCGs, rRNA, and tRNA genes are colored purple, green, and red, respectively. The arrows at the end of each feature indicate the transcriptional direction of the genes. GC content and GC-skew values were calculated using a 500 bp sliding window with a step of 1 bp. GC content was plotted using a black sliding window. Positive and negative values of GC-skew are plotted using green and purple sliding windows, respectively.

**Figure 3.**
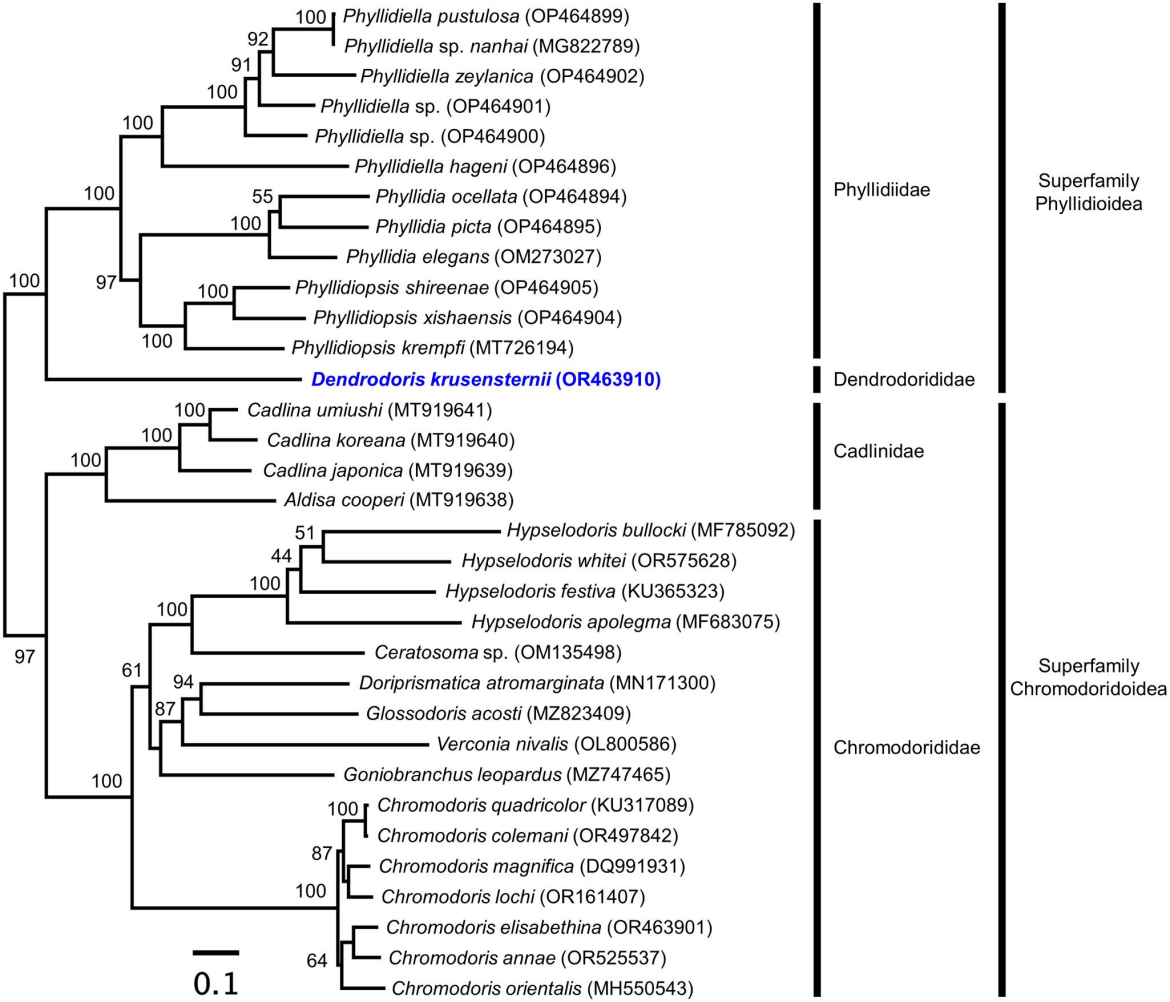
Phylogenetic tree inferred *via* ML using 13 PCGs from the mitochondrial genomes of six sea slugs, including *Dendrodoris krusensternii* (OR463910). Bootstrap support values based on 1,000 replicates are displayed on each node. Blue text denotes species whose sequences were reported for the first time in this study. A list of scientific names, accession numbers, and references for all sequences used in this phylogeny is provided in Table 1.

## Materials and methods

A specimen of *D. krusensternii* was collected in Yeosu, South Korea (34°44′31.46″N, 127°45′18.94″E) on November 9, 2022, at a depth between 0 and 1 m. A voucher specimen (MABIK MO00184346) has been deposited in ethyl alcohol (99.9%) in the National Marine Biodiversity Institute of Korea (Seunghyun Lee, hyun2@mabik.re.kr, Seocheon, South Korea). The specimen was morphologically identified as having a brown body, tubercles on the dorsum, and blue spots on the body (Nimbs and Smith 2021). Molecular identification revealed a 99.39% match to the *COX1* fragment (OQ744501; 630 bp). Total genomic DNA was extracted from the specimen using a DNeasy Blood & Tissue DNA kit (Qiagen, Hilden, Germany), from which a genomic library was constructed with paired-end reads using a QIAseq FX single-cell DNA library kit (Qiagen). The average insert size was 350 bp. The Novaseq X series 10B Reagent kit (300 cycles; Illumina Inc., CA, USA) was used for. Next-generation sequencing (NGS) was performed on the NovaSeq 6000 system (Illumina Inc.). This yielded a total of 37,906,316 paired-end raw reads. From the raw reads, 37,418,099 clean reads were obtained by trimming low-quality data and deleting adaptor sequences using Trim_Galore v0.6.1 (Krueger et al. 2021).

It is advantageous to use SPAdes when reference sequences are not available during de novo assembly (Milián-García et al. 2022), and we used SPAdes v3.15.5 (Prjibelski et al. 2020). Due to the high depth of coverage (mean read depth = 183,685), the assembly resulted in five contigs. These contigs were aligned to the mitochondrial genome of *Phyllidia elegans* (OM273027) using the National Center for Biotechnology Information (NCBI) Basic Local Alignment Search Tool (BLAST; Altschul et al. 1990). Based on the alignment, we ordered and merged the contigs to obtain a single circular mitochondrial genome sequence. The high levels of depth of coverage ensured the reliability of the sequence. Subsequently, the protein-coding genes (PCGs), rRNAs, and tRNAs of the assembled mitochondrial genome were annotated using the MITOS Webserver (Donath et al. 2019), and circular map were generated using CGView (Grant and Stothard 2008).

For phylogenetic analysis, we selected mitochondrial genome sequences from two superfamilies (Phyllidioidea and Chromodoridoidea). The Phyllidioidea superfamily was chosen as the ingroup, while Chromodoridoidea was the outgroup. Due to the limited availability of mitochondrial genome sequences for the family Dendrodorididae in GenBank, we selected Phyllidioidea as the ingroup and Chromodoridoidea as the outgroup for our phylogenetic analysis. Thirty-three published mitochondrial genome sequences were used for the phylogenetic analysis. Specifically, 12 and 21 published mitochondrial genome sequences were obtained from Phyllidioidea (ingroup) and Chromodoridoidea (outgroup), respectively (Table 1), from the NCBI GenBank (Sayers et al. 2022; accessed June 3, 2024).

**Table 1.** Mitochondrial genome sequences for phylogenetic analysis.

Group	Scientific name	Accession number	Reference
–	*Dendrodoris krusensternii*	OR463910	This study
	*Phyllidiella pustulosa*	OP464899	Liu et al. (2023)
	*Phyllidiella* sp. *nanhai*	MG822789	Liu et al. (2024)
	*Phyllidiella zeylanica*	OP464902	Unpublished
	*Phyllidiella* sp.	OP464901	Unpublished
	*Phyllidiella* sp.	OP464900	Unpublished
Ingroup	*Phyllidiella hageni*	OP464896	Unpublished
	*Phyllidia ocellata*	OP464894	Xiang et al. (2016a)
	*Phyllidia picta*	OP464895	Unpublished
	*Phyllidia elegans*	OM273027	Li et al. (2022)
	*Phyllidiopsis shireenae*	OP464905	Unpublished
	*Phyllidiopsis xishaensis*	OP464904	Unpublished
	*Phyllidiopsis krempfi*	MT726194	Kim et al. (2021)
	*Cadlina umiushi*	MT919641	Do et al. (2022)
	*Cadlina koreana*	MT919640	Do et al. (2022)
	*Cadlina japonica*	MT919639	Do et al. (2022)
	*Aldisa cooperi*	MT919638	Do et al. (2022)
	*Hypselodoris bullocki*	MF785092	Lin et al. (2019)
	*Hypselodoris whitei*	OR575628	Unpublished
	*Hypselodoris festiva*	KU365323	Karagozlu et al. (2016)
	*Hypselodoris apolegma*	MF683075	Lin et al. (2019)
	*Ceratosoma* sp.	OM135498	Unpublished
Outgroup	*Doriprismatica atromarginata*	MN171300	Do et al. (2019)
	*Glossodoris acosti*	MZ823409	Unpublished
	*Verconia nivalis*	OL800586	Do et al. (2022)
	*Goniobranchus leopardus*	MZ747465	Unpublished
	*Chromodoris colemani*	OR497842	Unpublished
	*Chromodoris quadricolor*	KU317089	Xiang et al. (2016b)
	*Chromodoris magnifica*	DQ991931	Medina et al. (2011)
	*Chromodoris lochi*	OR161407	Unpublished
	*Chromodoris elisabethina*	OR463901	Unpublished
	*Chromodoris annae*	OR525537	Lin et al. (2017)
	*Chromodoris orientalis*	MH550543	Yu et al. (2018)

The dataset utilized for the phylogenetic analysis comprised 13 PCGs from 34 species in two superfamilies. These genes were chosen because they are consistently present across metazoan mitochondrial genomes, encode essential proteins for cellular respiration, and are widely used in mitochondrial phylogenomics (Boore 1999; Bernt et al. 2013). This approach allowed for standardized comparisons while focusing on functionally relevant genomic regions. The selected genes were aligned using CLUSTAL Omega (Sievers et al. 2011) with Geneious Prime 2020.11.0. (Biomatters Ltd., Auckland, New Zealand). The substitution saturation test of the PCG dataset was evaluated using DAMBE v6.4.29 (Xia et al. 2003; Xia and Lemey 2009). The results of repeating the test for all individual gene alignments (Iss < Iss.c, *p* < 0.05) and the results for the entire PCG dataset (Iss = 0.363 < Iss.*c* = 0.818, *p* < 0.05; Table S1) indicated that the intersequence substitutions did not reach saturation. A maximum-likelihood (ML) tree was reconstructed using raxmlGUI 2.0 (Edler et al. 2021) with the GTR model and 1,000 bootstrap replicates. A model test was performed using the PAUP* plugin in Geneious Prime (Swofford 2002).

## Results

The mitochondrial genome of *D. krusensternii* (OR463910) has a total length of 14,361 bp, comprising 13 PCGs, 22 transfer RNA genes, and two ribosomal RNA genes (Figure 2). The overall nucleotide composition was 28.7% A, 14.8% C, 19.6% G, and 36.9% T. Most PCG start codons were ATG (*COX1*, *ND1*, *COX2*, *ATP8*, *ATP6*, *ND3*, *COX3*, and *ND2*). ATA was the start codon for *ND5*, *ND4L*, and *CYTB*. ATT was the start codon for *ND6*, while GTG was for *ND4*. In summary, ATT and GTG were alternative start codons for their respective genes, while ATG and ATA were the most common start codons. The most common stop codon was TAA (*COX1*, *ND6*, *ND4L*, *COX2*, *ATP8*, and *ATP6*) and TAG (*ND5*, *ND1, CYTB*, and *ND4*). The remaining three PCGs (*ND3*, *COX3*, and *ND2*) had an incomplete stop codon ‘T––’.

The results of ML tree (Figure 3) analysis indicated that *D. krusensternii*, which is included in Phyllidioidea, did not form a direct cluster with Phyllidiidae. Instead, it was placed as a sister taxon to the Phyllidiidae. Differences were observed in the length of mitochondrial genome annotations between *P. pustulosa* and *D. krusensternii.* Specifically, *ND4* differed by 87 bp, *ND5* by 57 bp, *12s* by 15 bp, and *16s* by 54 bp.

## Discussion and conclusion

Our sequencing results showed an unexpectedly high level of depth and coverage. In general, while high mapping reads should be accompanied by a specific mitochondrial enrichment strategy (Wu et al. 2023), no enrichment strategy was used in this study. Therefore, we assumed that mitochondria were abundant in the tissues of the specimen analyzed in this study. This anomaly may be due to the specific individuals (specimens). Alternatively, it is difficult to explain with our current understanding whether this is an anomaly for *D. krusensternii* at the species level, as studies have shown that molluscan mitochondria deviate from established genetic patterns (Ghiselli et al. 2021). Further research and closer examination are needed to explain these high depth and coverage results.

The start codons of *D. krusensternii* were expected to follow the invertebrate mitochondrial gene code (NCBI gene code 5; Ghiselli et al. 2021). ATT (*ND6*) and GTG (*ND4*) are often used as alternative start codons, including in molluscs (Dreyer and Steiner 2006). Other Nudibranchian mitochondrial genomes used in the dataset of phylogenetic analysis also terminated with an incomplete stop codon ‘T––’ in *ND3*, *COX3*, and *ND2* regions, consistent with our results. *ND3* had incomplete termination codons in six species (*Phyllidiella hageni*, *Phyllidiopsis krempfi*, *Phyllidiopsis xishaensis*, *Doriprismatica atromarginata*, *Hypselodoris festiva*, and *Chromodoris elisabethina*), and *COX3* in particular had incomplete termination codons in all species except two (*Ceratosoma* sp. and *Chromodoris quadricolor*). *ND2* had incomplete stop codons in 12 species of the Phyllidiella and Phyllidiopsis genera. Therefore, incomplete stop codons are considered common in the two superfamilies (Phyllidioidea and Chromodoridoidea) within Nudibranchia. The phylogenetic analysis species the three families (Phyllidiidae, Cadllinidae, and Chromodorididae) separately, consistent with previous findings of the nudibranchia mitochondrial genome (Wägele et al. 2003; Li et al. 2022).

Certain morphological studies suggested that the *Dendrodoris* was closer to *Chromodoris* (superfamily Chromodoridoidea) than Phyllidiella (superfamily Phyllidioidea; Brunckhorst 1993). Relatively recent studies have suggested that the Dendrodorididae and Phyllidiidae show disparate morphological characteristics (Wägele and Willan 2000; Nimbs and Smith 2021). However, the mitochondrial genome phylogeny results presented in this study render it challenging to ascertain which suggestion was correct. Although a few PCGs (*ND4* and *ND5*) and rRNAs (*12s* and *16s*) may vary in length (9–99 bp), the arrangement was not different. Morphological and molecular studies of variation in the genus *Dendrodoris* are increasingly being undertaken (Khanam and Kazmi 2016; Nimbs and Smith 2021; Gali-Camps et al. 2022). The mitochondrial genome sequence obtained in this study is expected to be a valuable genomic resource for further molecular studies on the evolution of the Dendrodorididae family.

## Supplementary Material

Supplementary information_final_1.docx

Figure S1 .tif

## Data Availability

The genome sequence data supporting the findings of this study are available in GenBank (https://www.ncbi.nlm.nih.gov/) under accession no. OR463910. The associated BioProject, SRA, and Bio-Sample numbers are PRJNA998227, SRR27032638, and SAMN36700722, respectively.
