# Supplementary material for: The complete mitochondrial genome of Dendrodoris krusensternii (Gastropoda, Nudibranchia, Dendrodorididae) from South Korea
Source: Mitochondrial DNA B Resour. 2024 Dec 5;9(12):1636–41. doi: 10.1080/23802359.2024.2435915 (PMC11622375; doi:10.1080/23802359.2024.2435915)
Supplement: Supplementary information_final_1.docx [file TMDN_A_2435915_SM4950.docx]

**Supplementary information**

The complete mitochondrial genome of *Dendrodoris krusensternii* (Gastropoda, Nudibranchia, Dendrodorididae) from South Korea

Seunghyun Lee^a^ and Seongjun Bae^a,*^

^a^*Department of Ecology and Conservation, Marine Biodiversity Institute of Korea*, *Seocheon 33662*, *South Korea*

ORCID : Seunghyun Lee (0009-0000-4553-5808) and Seongjun Bae (0000-0001-9998-0886)

^*^ Corresponding author: Seongjun Bae (silverto@naver.com)





Figure S1. Nucleotide coverage depth for *Dendrodoris krusensternii*.

Table S1. Saturation test results for the 13 PCGs in the mitochondrial genome of *Dendrodoris krusensternii*.

| PCG | Iss | Iss.c | p |
| --- | --- | --- | --- |
| ATP6 | 0.488 | 0.723 | 0.0427 |
| ATP8 | 0.638 | 0.706 | 0.0035 |
| COX1 | 0.244 | 0.776 | 0.0000 |
| COX2 | 0.481 | 0.724 | 0.0441 |
| COX3 | 0.324 | 0.731 | 0.0038 |
| CYTB | 0.300 | 0.756 | 0.0000 |
| ND1 | 0.343 | 0.742 | 0.0003 |
| ND2 | 0.508 | 0.745 | 0.0195 |
| ND3 | 0.506 | 0.688 | 0.0121 |
| ND4 | 0.617 | 0.772 | 0.0002 |
| ND4L | 0.463 | 0.682 | 0.0343 |
| ND5 | 0.419 | 0.781 | 0.0001 |
| ND6 | 0.493 | 0.700 | 0.0015 |
| Total 13 PCGs | 0.363 | 0.818 | 0.0000 |
